# The angiotensin II type I receptor contributes to impaired cerebral blood flow autoregulation caused by placental ischemia in pregnant rats

**DOI:** 10.1186/s13293-019-0275-1

**Published:** 2019-12-11

**Authors:** Junie P. Warrington, Fan Fan, Jeremy Duncan, Mark W. Cunningham, Babette B. LaMarca, Ralf Dechend, Gerd Wallukat, Richard J. Roman, Heather A. Drummond, Joey P. Granger, Michael J. Ryan

**Affiliations:** 1 Department of Physiology & Biophysics, University of Mississippi Medical Center, 2500 N. State Street, Jackson, MS 39216 USA; 2 Experimental and Clinical Research Center and Max-Delbrück Center for Molecular Medicine, and HELIOS Clinic Berlin, Berlin, Germany

**Keywords:** Pregnancy, Losartan, Cerebral blood flow autoregulation, AT1-AA

## Abstract

**Background:**

Placental ischemia and hypertension, characteristic features of preeclampsia, are associated with impaired cerebral blood flow (CBF) autoregulation and cerebral edema. However, the factors that contribute to these cerebral abnormalities are not clear. Several lines of evidence suggest that angiotensin II can impact cerebrovascular function; however, the role of the renin angiotensin system in cerebrovascular function during placental ischemia has not been examined. We tested whether the angiotensin type 1 (AT1) receptor contributes to impaired CBF autoregulation in pregnant rats with placental ischemia caused by surgically reducing uterine perfusion pressure.

**Methods:**

Placental ischemic or sham operated rats were treated with vehicle or losartan from gestational day (GD) 14 to 19 in the drinking water. On GD 19, we assessed CBF autoregulation in anesthetized rats using laser Doppler flowmetry.

**Results:**

Placental ischemic rats had impaired CBF autoregulation that was attenuated by treatment with losartan. In addition, we examined whether an agonistic autoantibody to the AT1 receptor (AT1-AA), reported to be present in preeclamptic women, contributes to impaired CBF autoregulation. Purified rat AT1-AA or vehicle was infused into pregnant rats from GD 12 to 19 via mini-osmotic pumps after which CBF autoregulation was assessed. AT1-AA infusion impaired CBF autoregulation but did not affect brain water content.

**Conclusions:**

These results suggest that the impaired CBF autoregulation associated with placental ischemia is due, at least in part, to activation of the AT1 receptor and that the RAS may interact with other placental factors to promote cerebrovascular changes common to preeclampsia.

## Background

Preeclampsia is a complex syndrome of pregnancy that can negatively impact multiple organ systems and promote poor maternal and fetal outcomes. The brain is among the organs impacted during preeclamptic pregnancies. Preeclampsia increases the risk for maternal encephalopathies, seizure, edema, and stroke both in the peripartum period and even years into the postpartum period (reviewed in [1]). Indeed, 40% of maternal deaths that result from preeclampsia are related to cerebrovascular complications [2]. While the underlying mechanisms responsible for the increased cerebrovascular risk during preeclampsia remain incompletely understood, there is evidence both in patients and in experimental animal models of preeclampsia suggesting that impaired autoregulation of cerebral blood flow (CBF) may be a factor [3–5]. However, the mechanisms responsible for the impaired autoregulation of CBF during preeclampsia have yet to be elucidated.

One potential system that may be involved is the renin-angiotensin system (RAS). During normal pregnancies, the RAS is activated and plays a prominent role in the expansion of the extracellular fluid volume that accompanies normal pregnancy [6]. Blood pressure is typically unchanged or lower under these circumstances because of a reduced sensitivity to angiotensin II (AngII) that occurs during pregnancy [7]. However, in preeclamptic pregnancies, AngII sensitivity is increased and may be an important factor associated with the pathophysiology [8].

Evidence suggests that AngII causes cerebrovascular dysfunction that is attenuated by blockade of the angiotensin type 1 (AT1) receptor [9, 10]. In addition, AngII has been reported to directly alter CBF autoregulation in a sex specific manner. For example, relative to males, the ability of AngII to impair cerebral vascular responses to the whisker barrel reflex are blunted in females, and this protection results in part from the presence of estrogens [11]. While there is evidence that AT1 receptor activation leads to cerebrovascular dysfunction, the contribution of angiotensin receptors to impaired CBF autoregulation during pregnancy and preeclampsia remains unclear.

In the present study, we utilized an established experimental model of placental ischemia that mimics several characteristics of human preeclampsia to examine the role of AT1 receptors in CBF autoregulation. We previously reported that reducing uterine perfusion in the pregnant rat causes placental ischemia and leads to a marked impairment of CBF autoregulation [5]. In order to examine the role of AT1 receptors, we first utilized pharmacological blockade with losartan in placental ischemic rats. Next, we infused an agonistic AT1 receptor autoantibody (AT1-AA) into pregnant rats that is reportedly increased in preeclamptic women [12] and enhances AT1 receptor sensitivity [13]. The results of the present study suggest that the AT1 receptor partially contributes to impaired CBF autoregulation in placental ischemic rats.

## Methods

### Animals

Time-pregnant CD rats (a sub-strain of Sprague Dawley rats) were obtained from Charles River Laboratories and maintained in the Laboratory Animal Facilities at the University of Mississippi Medical Center and maintained in controlled temperature, humidity, and 12 h light/dark conditions. Rats had continuous access to food and water and were housed in pairs until day of surgery, after which, rats were singly housed. All animal protocols were approved by the Institutional Animal Care and Use Committee (IACUC) at UMMC before the experiments were conducted.

### Placental ischemia induction and losartan treatment

On gestational day (GD) 14, rats were weighed and weight-matched to sham or RUPP groups. Under isoflurane anesthesia, silver clips were surgically placed on the abdominal aorta, below the kidneys, and on both branches of the uterine arteries before the first pup. This procedure leads to reduction in utero-placental perfusion pressure (RUPP) and placental ischemia. Rats in the sham group were subjected to similar surgical interventions with abdominal incision and exteriorization of pups without clip placement. Carprofen (5 mg/kg) was administered as a pre- and post- surgical analgesic. Losartan (American Health Packaging, Columbus, OH) was administered via drinking water to pregnant rats from GD 14. Water intake was recorded daily from GD 14 to 19. Based on water intake, the rats in the sham group received 16.1 ± 0.9 mg/kg/day while rats in the RUPP group received 17.5 ± 1.3 mg/kg/day losartan (*p* = 0.50).

### Mean arterial pressure and other characteristics

On GD 18, a catheter was surgically implanted in the left carotid artery under isoflurane anesthesia. Blood pressure was measured in conscious rats in restrainer cages the morning of GD 19 via a carotid catheter connected to a pressure gauge and PowerLab setup (ADInstruments) as previously described [5]. Data were recorded in real-time following a 30 min acclimation period using LabChart software. Mean arterial pressure was recorded over a 30 min period. At the conclusion of the study, rats were anesthetized with isoflurane, and an abdominal incision was made in order to exteriorize the utero-placental unit. The number of live and resorbed pups was counted. Rats with no surviving pups (100% resorptions) were not considered as pregnant and were excluded from analysis (*n* = 12 in RUPP, *n* = 4 in RUPP+losartan).

### Cerebral blood flow autoregulation

On GD 19, a separate group of rats were anesthetized using ketamine (30 mg/kg, i.m.) and inactin (50 mg/kg, i.p.). Once an anesthetic plane was achieved, rats were instrumented with femoral vein catheters (for infusion of saline and/or phenylephrine), a carotid catheter (for continuous recording of blood pressure), and tracheal tubing (PE-240, for ventilation and monitoring of exhaled carbon dioxide). Cranial windows were created by thinning the skull until the brain surface vessels were visible, without puncturing the underlying dura. A 4 mm × 4 mm closed cranial window was created over the parietal cortex, and probe holders were affixed to the skull. The probes were in the region of the middle cerebral artery. Respiration rate was set based on the body mass of the rat according to manufacturer’s directions and was modified to maintain CO_2_ levels within physiological range (PhysioSuite with MouseVent, Kent Scientific). End-tidal CO_2_ has been shown to be a good indicator of arterial pCO_2_ [14]. Phenylephrine (50 μg/mL in saline) was infused via femoral vein catheter to induce graded increases in blood pressure. Baseline regional CBF was measured, and mean arterial pressure (MAP) was then elevated in steps of 20 mmHg up to 190 mmHg by graded intravenous infusion of phenylephrine (0.5–5 μg/min). CBF was monitored and recorded at each level of MAP using laser Doppler flowmetry (Perimed). We previously published using this method [5]. Autoregulatory index was calculated as a ratio of the change in CBF and the change in MAP. An autoregulatory index of 1 represents complete loss of autoregulation while 0 represents perfect autoregulation.

### AT1-AA infusion

Mini-osmotic pumps (Alzet, model 2001), containing either an agonistic autoantibody to the AngII type 1 receptor (AT1-AA) or vehicle (saline), were placed in peritoneal cavity in another group of pregnant rats on GD 12. The AT1-AA has been implicated in the pathogenesis of preeclampsia both in humans and experimental models [12, 15, 16]. The antibody was used at a 1:40 dilution of the original purified stock as previously described [13]. On GD 19, rats were prepared for measurement of MAP and assessment of CBF autoregulation as described above. In a separate group of animals, AT1-AA or vehicle was infused as above. Rats were euthanized on GD 19, and brains were collected to assess water content using the wet:dry weight ratio as previously described [17].

### Statistical analysis

Differences in CBF autoregulation curves for the Losartan study were analyzed using a mixed effects model three-way analysis of variance (ANOVA) with MAP as a repeated measure and two (factors: group: sham versus RUPP, and treatment: vehicle versus losartan). Similarly, the curves for the AT1-AA study were analyzed using 2-way repeated measures ANOVA with one factor (vehicle versus AT1-AA antibody) and MAP as a matching variable followed by Holm-Sidak post-hoc test. For the losartan treatment, differences in pregnancy outcome and mean arterial pressure were analyzed using 2-way ANOVA with two factors (group: Sham versus RUPP, and treatment: vehicle versus Losartan). For pregnancy outcomes in the AT1-AA study, an unpaired *t* test was used. A *p* value of less than 0.05 was considered statistically significant. All statistical analyses and figures were calculated and generated using GraphPad Prism (version 7.02).

## Results

### Impact of AT1 receptor blockade on pregnancy outcomes

A summary of pregnancy outcomes for rats treated with losartan is provided in Table 1. Placental ischemia resulted in a decrease in dam body mass which was not prevented in losartan treated animals (*p* value for interaction = 0.863). Losartan did not prevent the reduction in live pups typically induced by placental ischemia (*p* value for interaction = 0.769) nor did it reduce the number of fetal resorptions in dams that remained pregnant for the duration of the study (*p* value for interaction = 0.828). However, fewer losartan treated placental ischemic rats had complete (100%) resorption of all pups (*n* = 4) compared with vehicle-treated placental ischemic rats (*n* = 12). There was no effect of RUPP or losartan treatment on pup (*p* value for interaction = 0.106) or placental weight (*p* value for interaction = 0.058). Thus, the general pregnancy outcomes were not altered in animals treated with losartan.

**Table 1:** Pregnancy outcomes in response to placental ischemia and losartan treatment

Characteristics	Sham (*N* = 11)	RUPP (*N* = 17)	Sham + Los (*N* = 10)	RUPP + Los (*N* = 17)
Body mass (g)	334.4 ± 6.5	298.2 ± 6.7^*^	337.0 ± 4.8†	300.7 ± 5.5^*‡^
No. of live pups	13 ± 0	7 ± 1^*^	13 ± 1†	7 ± 1^*‡^
No. of resorptions	0 ± 0	7 ± 1^*^	1 ± 0†	7 ± 1^*‡^
Pup weight (g)	2.29 ± 0.05	2.25 ± 0.04	2.49 ± 0.07	2.27 ± 0.06
Placenta weight (g)	0.46 ± 0.02	0.50 ± 0.02	0.51 ± 0.02	0.47 ± 0.02

^*^p < 0.05 vs. Sham

^†^*p* < 0.05 vs. RUPP

^‡^p < 0.05 vs. Sham + Los

### Losartan treatment prevents placental ischemia-induced hypertension

**Fig. 1:**
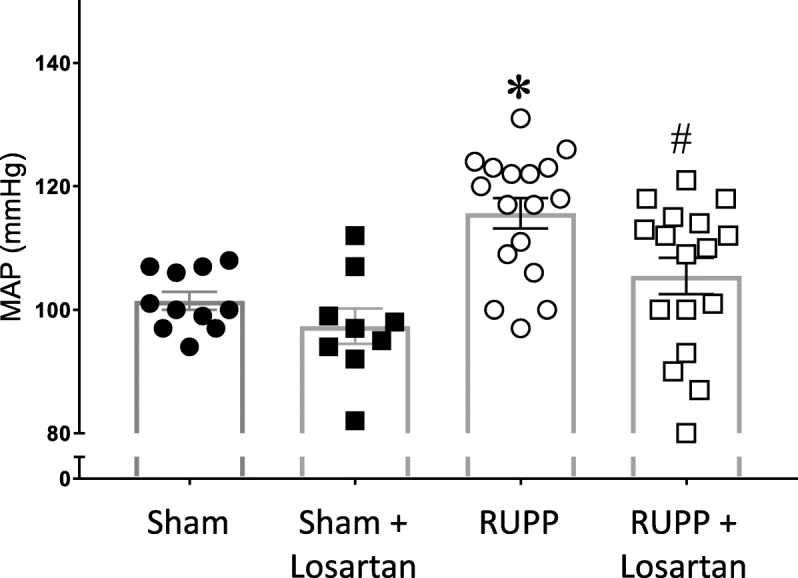
Losartan prevents placental ischemia-induced increase in blood pressure. Blood pressure was measured in conscious rats on GD19 via indwelling carotid artery catheter. Data points for each rat are shown along with the Mean ± SEM. *p < 0.05 compared with Sham, #*p* < 0.05 compared with RUPP using 2-way ANOVA, with Holm-Sidak post hoc test. *N* = 9–17 rats per group/treatment. RUPP–reduced uterine perfusion pressure

Compared with sham pregnant rats, placental ischemia caused a significant increase in mean arterial pressure (116 ± 2 in RUPP vs. 101 ± 1 mmHg in Sham; *p* < 0.05, Fig. 1). Losartan treatment did not alter blood pressure in the Sham group (97 ± 3 mmHg), but it prevented RUPP-induced increases in MAP (105 ± 3 mmHg, *p* < 0.05). There was no significant interaction between RUPP surgery and losartan treatment (*p* = 0.239).

### Losartan treatment prevents placental ischemia-induced CBF autoregulation impairment

**Fig. 2:**
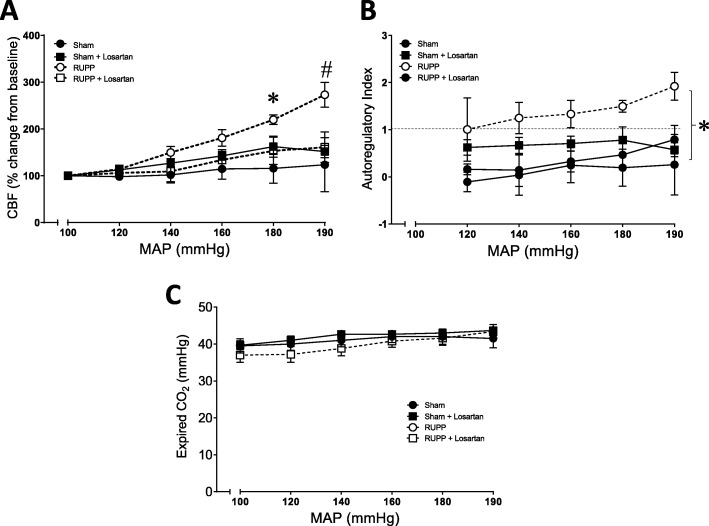
AT1R blockade prevents placental ischemia-induced impairments in CBF autoregulation. **a** Blood pressure was plotted against % change in CBF for Sham (*n* = 4), RUPP (*n* = 7), Sham + losartan (*n* = 4), and RUPP+losartan (*n* = 3) groups. *p < 0.05 compared with Sham. #*p* < 0.05 compared to Sham, Sham+losartan, RUPP+losartan. **b** Autoregulatory Index was plotted against changes in MAP. Dashed horizontal line (AI = 1) corresponds to the complete loss of autoregulation. *p < 0.05 RUPP vs. Sham group effect. Data represent mean ± SEM. Statistics calculated using three way repeated measures ANOVA

Changes in CBF in response to increased MAP were compared in Sham rats, Sham rats treated with losartan, RUPP, and RUPP rats treated with losartan. There was a significant interaction between treatments and groups (*p* = 0.013), the effect of MAP × group × treatment was significantly different (0.0267), and there is a main effect of losartan treatment on CBF (*p* < 0.014). At 180 mmHg and above, the relative increase in CBF was significantly greater in RUPP vehicle-treated vs. Sham rats receiving vehicle. However, losartan treatment prevented this RUPP-induced increase in CBF (*p* < 0.01 vs. RUPP; Fig. 2a). Autoregulatory Index was calculated for each MAP step and plotted in Fig. 2b. There was a significant effect of treatment on autoregulatory index (*p* = 0.045) calculated as the percent change in CBF divided by the change in MAP. An index > 1, as shown in the RUPP animals, is indicative of markedly impaired autoregulatory function with a compliant vasculature. Autoregulatory index was < 1 in all sham controls and in RUPP animals treated with losartan. There was a significant effect of group × treatment on CBF (*p* = 0.018). Expired carbon dioxide was recorded throughout the study because small increases in CO2 significantly increase CBF. There was a main effect of group and treatment on CO_2_ (*p* < 0.0001); however, multiple comparisons analysis did not yield any statistical differences across the groups. While group × treatment effect was significantly different (*p* < 0.0001), there was no significant effect of MAP × group × treatment (*p* = 0.446) (Fig. 2c). Brain water content, a marker of cerebral edema, was not different between sham and RUPP rats treated with either vehicle or losartan (Table 3).

### AT1-AA infusion into pregnant rats impairs CBF autoregulation

**Fig. 3:**
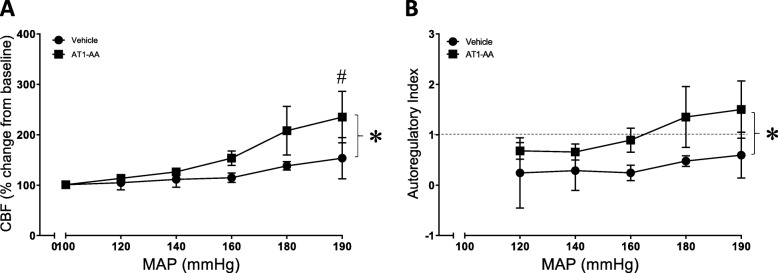
Chronic infusion of AT1-AA into pregnant rats induced impaired CBF autoregulation. **a** Changes in CBF were plotted against mean arterial pressure in vehicle—(*n* = 3) or AT1-AA—(*n* = 4) infused rats. **b** Autoregulatory index was calculated for each pressure step and plotted against changes in MAP. Dashed horizontal line (AI = 1) corresponds to the complete loss of autoregulation. Data represent mean ± SEM. *p < 0.05 compared with vehicle. #*p* < 0.05 AT1-AA at 190 mmHg vs AT1-AA at 100 mmHg. Statistics were calculated using two-way repeated measures ANOVA

An agonistic AT1 receptor autoantibody has been reported in women with preeclampsia [12]. The AT1-AA has been proposed to enhance the sensitivity of AT1 receptors to the vasoconstrictor actions of AngII [13]. Therefore, we examined whether infusion of the AT1-AA into normal pregnant rats would impair CBF autoregulation similar to what occurs in placental ischemic rats. Table 2 shows the pregnancy outcomes for pregnant rats that received either a vehicle minipump or an AT1-AA minipump. Chronic infusion of the AT1-AA did not alter body weight, pup numbers, pup weight, or placental weight. Mean arterial pressure was 105 ± 3 mmHg (*n* = 11) in the AT1-AA infused pregnant rats compared with 98 ± 3 mmHg (*n* = 7) in the vehicle infused pregnant rats and was not different between the groups. The infusion of the AT1-AA into pregnant rats did not alter brain water content (Table 3). In a separate group of animals, AT1-AA and vehicle infused pregnant rats were anesthetized and intubated on GD 19 for assessment of CBF autoregulation (Fig. 3). The infusion of the AT1-AA significantly impaired the CBF response to increasing arterial pressure. Consistent with impaired autoregulatory function, autoregulatory index stayed < 1 in the normal pregnant rats, but was > 1 in AT1-AA infused rats at the highest pressures.

**Table 2:** Pregnancy outcomes in response to AT1-AA infusion

Characteristics	Vehicle (*N* = 7)	AT1-AA (*N* = 11)
Body mass (g)	312.7 ± 9.4	308.7 ± 6.8
No. of live pups	12 ± 0	11 ± 1
No. of resorptions	0 ± 0	0 ± 0
Pup weight (g)	2.43 ± 0.08	2.40 ± 0.07
Placenta weight (g)	0.59 ± 0.03	0.59 ± 0.04

**Table 3:** Brain water content [(wet-dry weight/wet weight)*100]

Experimental group	Anterior brain (%)	Posterior brain (%)	*n*
Sham	79.4 ± 0.1	78.3 ± 0.2	7
RUPP	79.3 ± 0.1	78.0 ± 0.1	12
Sham+Los	79.1 ± 0.2	78.0 ± 0.2	5
RUPP+Los	78.5 ± 0.6	78.2 ± 0.2	8
Vehicle	79.9 ± 0.1	78.2 ± 0.1	7
AT1-AA	79.4 ± 0.6	78.0 ± 0.4	12

## Discussion

Cerebral complications are now considered a diagnostic symptom of preeclampsia when accompanied by new onset hypertension [18]. In addition, clinical studies in preeclamptic patients point to vascular complications as a major contributor to cerebral symptoms. Indeed, approximately 40% of (pre)eclampsia-related deaths can be attributed to cerebrovascular events [2]. Therefore, continued investigation of the potential underlying mechanisms that contribute to the cerebral consequences of preeclampsia is required. The present study builds upon the current literature by making the following important advances. (1) The AT1 receptor contributes to the pathogenesis of hypertension and to the impaired CBF autoregulatory function in placental ischemic rats. (2) An agonistic autoantibody to the AT1 receptor (AT1-AA), which has been implicated in the pathogenesis of preeclampsia by increasing AT1 receptor sensitivity, impairs CBF autoregulation during pregnancy. Taken together, these data suggest that activation of the AT1 receptor may have important functional consequences in the cerebral vasculature during pregnancy, perhaps in part through a mechanism involving an agonistic AT1-AA.

During a normal pregnancy, components of the RAS are increased including renin, angiotensinogen, and aldosterone [6]. The increase in RAS during normal pregnancy is an important physiological adaptation that promotes the normal extracellular fluid volume expansion. Despite an increased circulating RAS, vascular sensitivity to AngII is decreased such that greater than 2× the normal amount is required to induce the same level of vasoconstriction [19]. During a preeclamptic pregnancy, considerable evidence in both humans and experimental models suggest that vascular sensitivity to AngII is increased. For example, a study from 1973 by Gant et al. showed that vascular responses to AngII were enhanced in primigravid women prior to the development of pregnancy induced hypertension [20]. There is also evidence for increased adrenal sensitivity to AngII during pregnancies complicated by preeclampsia [20]. These data suggest a role for the AT1 receptor in the pathogenesis of preeclampsia. Experimentally, the potential importance of the AT1 receptor was demonstrated by incubating human umbilical vein endothelial cells with serum from the RUPP model of placental ischemia. In that study, serum from rats with placental ischemia increased human umbilical vein endothelial cell production of endothelin, an effect blocked by an AT1 receptor inhibitor [21]. Moreover, a separate study reported that AT1 receptor blockade in the RUPP model of placental ischemia attenuates the hypertension [15]. The data in the present study are consistent with an important role for the RAS in the pathogenesis of preeclampsia, and replicates earlier findings showing that blood pressure in placental ischemic rats is sensitive to AT1 receptor blockade.

Chronic AngII-induced hypertension directly causes hypertrophic inward remodeling of the cerebral vasculature which can negatively impact brain perfusion and shift the range of autoregulation of CBF to higher pressures (reviewed in [22–25]). However, there is an important sexually dimorphic response to chronic AngII with blunted cerebral blood flow responses to AngII in female mice compared with males suggesting that the cerebral vasculature is regulated by AngII differently in males and females [11]. While the role of AT1 receptors in cerebral vascular function is generally well known, as is their contribution to preeclampsia, much less is understood about the contributions of the AT1 receptors on the impaired CBF autoregulation during preeclampsia. During a normal pregnancy, AT1 receptor expression is reduced in the cerebral vasculature [26]. This reduction in receptor expression may contribute to the protection against cerebral vascular inward remodeling during hypertensive pregnancies. For example, both nitro-l-arginine–induced hypertensive and Dahl S hypertensive rats are protected against cerebral vascular remodeling during pregnancy [27, 28]. Consistent with these findings, we previously reported that cerebral vessels from placental ischemic rats do not undergo inward remodeling [17]. In addition, we showed that CBF autoregulation is impaired in the placental ischemic model caused by RUPP [5]. The present study confirms and extends upon these findings by showing that the impaired autoregulatory function can be ameliorated with AT1 receptor blockade, thus suggesting a mechanistic role for the AT1 receptors.

The cellular pathway by which AT1 receptor blockade during placental ischemia improves autoregulatory function remains unclear, although one can speculate as to possibilities. For example, it has been previously shown that treatment with losartan increases the amount of AngIV produced which ultimately leads to an increased AngIII production and activation of the vascular protective AT2 receptor [29]. Another possibility may relate to evidence showing that AngII reduces immunolabeled βENaC in renal vascular smooth muscle [30]. This is potentially important because βENaC is an essential component of the vascular mechanosensor that contributes to the myogenic response [31], and we previously showed that βENaC protein expression is lower in cerebral arteries from placental ischemic rats when compared with normal pregnant rats [17]. Therefore, if cerebral vascular responses to AngII mirror those in the renal vasculature, it may be that angiotensin mediated downregulation of βENaC is a contributing mechanism. Another possibility by which AngII can induce impaired vascular reactivity is through generation of reactive oxygen species (ROS) (reviewed extensively in [22]). Thus, it is possible that increased vascular ROS production in the setting of placental ischemia, primes the vasculature for increased injury in response to endogenous and exogenous levels of AngII or AT1-AA. This possibility will be addressed in future studies.

We also considered the possibility of a role for the AT1-AA. AT1-AA was first reported in women with preeclamptic pregnancies by Wallukat et al. [12]. AT1-AA is thought to act by increasing the sensitivity of the AT1 receptor; however, its role in the cerebral vasculature has not been examined until now. Previously published work from the LaMarca laboratory reports that placental ischemia increases the production of AT1-AA in rats [15]. Moreover, infusion of the AT1-AA into pregnant rats replicates features of the placental ischemic model including the hypertension [32]. In the present study, infusion of the AT1-AA did not increase MAP. This result is more in line with the work of Wenzel et al. who generated an activating AT1 receptor antibody (AT1-AB) with the same chronotropic activity as the AT1-AA, but by itself did not cause changes in MAP in pregnant rats [33]. The reason for the difference in blood pressure response is not clear; however, it may be due to genetic differences in the rats used in the studies. The original studies utilized Sprague Dawley (SD) rats, whereas the present study was performed in CD rats, a sub-strain of SD rats. Interestingly, infusion of the AT1-AA caused impaired CBF autoregulation in pregnant rats, suggesting that it may play a mechanistic role in the cerebral vascular changes during preeclampsia independent of changes in blood pressure.

## Conclusions

Although cerebrovascular events contribute significantly to the morbidity and mortality in patients with preeclampsia, the mechanisms involved are not clear. In this study, we present evidence that blockade of the AT1 receptor with losartan following placental ischemia prevents the hypertension and loss of CBF autoregulation associated with preeclampsia. It remains to be determined whether the lowering of blood pressure or blockade of the receptor is responsible for the improvement. While our data support the idea that the AT1 receptor is a promising therapeutic target for the treatment of cerebrovascular abnormalities associated with placental ischemia and preeclampsia, RAS inhibition is contraindicated during pregnancy. In addition, we cannot rule out the possibility that AngII or AT1-AA are not directly impacting cerebral vascular function, but rather having an indirect effect in the brain due to actions in other organs. Therefore, tissue specific therapies that target the AT1 receptor or the AT1-AA may be useful as potential therapeutic agents.

## Data Availability

The datasets used and/or analyzed during the current study are available from the corresponding author on reasonable request.
